# Biologic predictors of clinical improvement in rituximab-treated refractory myositis

**DOI:** 10.1186/s12891-015-0710-3

**Published:** 2015-09-17

**Authors:** Ann M. Reed, Cynthia S. Crowson, Molly Hein, Consuelo Lopez de Padilla, Jeannette M. Olazagasti, Rohit Aggarwal, Dana P. Ascherman, Marc C. Levesque, Chester V. Oddis, the RIM Study Group

**Affiliations:** Division of Pediatric Rheumatology, Department of Pediatrics, Duke University School of Medicine, 201 Trent Drive, Durham, NC 27710 USA; Division of Rheumatology, Mayo Clinic, Rochester, MN USA; Division of Health Sciences Research, Mayo Clinic, Rochester, MN USA; Division of Rheumatology, University of Pittsburgh, Pittsburgh, PA USA; Division of Rheumatology, University of Miami Miller School of Medicine, Miami, FL USA

## Abstract

**Background:**

To examine the longitudinal utility of a biomarker signature in conjunction with myositis autoantibodies (autoAbs) as predictors of disease improvement in refractory myositis patients treated with rituximab.

**Methods:**

In the RIM Trial, all subjects received rituximab on 2 consecutive weeks. Using start of treatment as baseline, serum samples (*n* = 177) were analyzed at baseline and after rituximab with multiplexed sandwich immunoassays to quantify type-1 IFN-regulated and other pro-inflammatory chemokines and cytokines. Biomarker scores were generated for the following pathways: type-1 IFN-inducible (IFNCK), innate, Th1, Th2, Th17 and regulatory cytokines. Myositis autoAbs (anti-synthetase *n* = 28, TIF-γ *n* = 19, Mi-2 *n* = 25, SRP *n* = 21, MJ *n* = 18, non-MAA *n* = 24, unidentified autoantibody *n* = 9, and no autoantibodies *n* = 33) determined by immunoprecipitation at baseline, were correlated with outcome measures. Kruskal-Wallis rank sum tests were used for comparisons.

**Results:**

The mean (SD) values for muscle disease and physician global disease activity VAS scores (0–100 mm) were 46 (22) and 49 (19). IFNCK scores (median values) were higher at baseline in subjects with anti-synthetase (43), TIF1-γ (31) and Mi-2 (30) compared with other autoAb groups (*p* < 0.001). At 16 weeks after rituximab, anti-synthetase and Mi-2 autoAb positive subjects and non-MAA had a greater improvement in IFNCK scores (− 6.7, − 6.1 and −7.2, *p* < .001). Both IFNCK high scores (>30) and autoAb group (Mi-2, non-MAA, and undefined autoantibody) demonstrated the greatest clinical improvement based on muscle VAS (muscle-interaction *p* = 0.075).

**Conclusion:**

Biomarker signatures in conjunction with autoAbs help predict response to rituximab in refractory myositis. Biomarker and clinical responses are greatest at 16 weeks after rituximab.

## Background

The idiopathic inflammatory myopathies (IIMs) are a heterogeneous group of chronic acquired disorders characterized by muscle inflammation and proximal muscle weakness. These include adult polymyositis (PM), and both adult and juvenile dermatomyositis (DM) [1]. Both manual muscle testing (MMT) and serum levels of muscle enzymes have been used as markers of disease activity for IIM [2]. However, MMT may sometimes be inaccurate since muscle strength may be impaired by disease damage such as chronic scarring, fibrosis or atrophy rather than ongoing disease activity; in other cases weakness cannot be detected in some patients [2]. Similarly, muscle enzyme levels may be inadequate since they are not specific, may decrease even with ongoing muscle inflammation and may be elevated in non-inflammatory myopathies and in denervating conditions [3]. Furthermore, levels of muscle enzymes may be normal in cases of advanced IIM due to fatty replacement of muscle tissue and in patients with decreased muscle mass [3].

Detection of myositis-specific autoantibodies (MSAs) can also be helpful in the proper clinical scenario. Examples of MSAs are autoantibodies directed against aminoacyl t-RNA synthetase (anti-syn); the best known is anti-Jo-1. Anti-Jo-1 is typically found in patients with antisynthetase syndrome, which is characterized by myositis, interstitial lung disease (ILD), polyarthritis, Raynaud’s phenomenon, and mechanic’s hands [3]. Therefore, the detection of anti-Jo-1 has important prognostic value. While very specific for IIM, anti-Jo-1 has a low sensitivity, and is only present in 20–30 % of PM patients and even less frequently in DM patients (5–10 %) [4]. Since we currently lack adequate indicators for disease activity, disease prognosis, and response to treatment, newer, more sensitive and responsive biomarkers are being sought.

Recently, many biomarkers have been identified in IIM pathogenesis, in particular the pro-inflammatory cytokine IL-6 and type 1 interferon (IFN) regulated genes. IL-6 modulates the innate and adaptive immune responses, stimulates tissue inflammation, and has both B- and T-cell differentiation activity [5]. Type 1 IFNs are important in up-regulating MHC class I expression, stimulating activated T cells, activating natural killer cells, and influencing dendritic cell maturation [5]. The use of cytokines such as IL-6 and type I IFN signatures has been studied prospectively by Reed et al. who determined that type 1 IFN peripheral blood gene “scores,” chemokine signatures as well as levels of IL-6, IL-8, and TNF may serve as sensitive and responsive longitudinal biomarkers of change in disease activity in juvenile and adult DM [2].

Management goals for IIM include eliminating organ inflammation and preventing disease complications to reduce morbidity and restore quality of life. Corticosteroids are the standard first-line therapy alone or in combination with immunosuppressive agents [6]. Unfortunately, many patients are refractory to corticosteroids and immunosuppressive agents, and therefore newer modes of therapy are currently being studied. B cell depletion (BCD) therapy with rituximab, which has been used for many years to treat B-cell lymphomas, has recently gained popularity in the treatment of autoimmune diseases. The recently published Rituximab in Myositis (RIM) trial assessed the effectiveness of rituximab in refractory adult PM and adult and juvenile DM, using validated measures of myositis disease activity and damage, a consensus-driven definition of improvement, and a unique randomized placebo-phase trial design [1]. Eighty-three percent of the enrolled subjects met the International Myositis Assessment and Clinical Studies Group preliminary definition of improvement by the end of the trial. Furthermore, the addition of rituximab provided a significant steroid-sparing effect between the start and conclusion of the trial [1].

While biologic therapies such as rituximab have resulted in improved treatment regimens for autoimmune diseases, the use of biologic therapies in clinical practice may be limited by concerns over cost. Therefore it is important to study which patients are most likely to benefit from biologics, not only to prevent unnecessary costs but to also prevent adverse effects. Currently there are no known biomarkers to help predict clinical improvement with rituximab in patients resistant to standard immunosuppressive therapy. Data presented by Lopez de Padilla et al., which analyzed this same RIM trial population, suggested that serum cytokines play an important role in the pathogenesis of myositis by initiating and perpetuating various cellular and humoral autoimmune processes. Using multiplexed sandwich immunoassays, they revealed that the interferon chemokine (IFNCK) and innate cytokine scores before treatment may help to identify refractory myositis patients responsive to rituximab [7].

Similarly Aggarwal et al. [8] recently used data from the RIM trial to identify laboratory predictors of clinical response in myositis patients treated with rituximab. They analyzed the effect of diverse variables such as myositis autoantibodies at baseline (anti-synthetase, −Mi-2, −SRP, −TIF1-γ, −MJ, or other autoantibodies) as predictors of the time to improvement. They found that anti-synthetase and anti-Mi-2 autoantibodies strongly predicted improvement in rituximab-treated refractory myositis patients [8]. Consequently, taking into account these recent findings, we examined the longitudinal utility of a biomarker signature in conjunction with myositis autoantibodies (autoAbs) as biomarkers of disease outcome in refractory myositis patients treated with B cell depletion (BCD).

## Methods

### Subjects and study design

#### Subjects

This study enrolled 200 subjects with refractory adult (*n* = 76) and juvenile DM (*n* = 48) and adult PM (*n* = 76). All subjects were part of a previously reported multicenter clinical trial, ‘Rituximab in Myositis (RIM) study’ [1].

This study was carried out in accordance with research protocols approved by Institutional Review Boards of the Mayo Clinic. Patients and legal guardians signed informed consent and/or assent, and samples were de-identified in the laboratory.

#### Study design

As previously reported [1], the RIM study used a randomized, double-blind, placebo-phase design of intravenous rituximab in which refractory subjects were randomized to either an ‘early-start arm (rituximab at weeks 0/1, placebo at weeks 8/9) or ‘late-start arm’ (placebo at weeks 0/1, rituximab at weeks 8/9); therefore all subjects received rituximab.

#### Clinical assessment

Clinical assessment and outcome measures were evaluated using a core set of measures (CSMs) described by the International Myositis Assessment and Clinical Studies group (IMACS) and used in myositis clinical trials, including physician global and extramuscular disease activity scores using a composite score (based on the investigator’s composite assessment of disease activity on the constitutional, cutaneous, skeletal, gastrointestinal, pulmonary, and cardiac scales of the Myositis Disease Activity Assessment Tool [MDAAT]. All the measures including physician and patient global assessments of disease activity and muscle strength were rated using 100 mm-Visual Analog Scale (VAS) scores (0–100), with higher scores indicating severe disease activity. All study participants had their disease activity assessed and blood samples collected at the time of entry to the study and at follow up visits.

#### Autoantibody assessment

MSAs were performed at the rheumatology research laboratory at the University of Pittsburgh using immunoprecipitation techniques as previously described [1]. Patients were grouped based on the presence of MSAs at baseline into the following groups:anti-synthetase, −Mi-2, −SRP, −TIF1-γ, −MJ, or other autoantibodies, as well as no autoantibodies and undefined autoantibodies. Other autoantibodies include those individuals with myositis-associated autoantibodies not listed previously; no autoantibodies includes those who had no autoantibodies detected; and undefined autoantibodies include those with autoantibodies that are not able to be defined.

#### Measurement of serum cytokines and chemokines

As previously described in detail in Reed et al., serum was isolated from blood drawn and, consequently, multiplexed sandwich immunoassays (Meso Scale Discovery, Rockville, MD) were used to quantitate the serum levels of IFN regulated chemokines, IFN-α, IFN-γ, and the serum levels of inflammatory cytokines [9, 8]. A composite IFN-regulated score was generated based on serum levels of 3 IFN-regulated chemokines (IP-10, I-TAC and MCP-1), Th1-(IFN-γ, TNFα, IL2), Th2-(IL-4, IL-5, IL-10, IL-12p70, IL-13), TH17-(IL-6, IL-17, IL-1β), innate cell-related cytokines (IFN-α, MCP-2, MIG, MIP-1β, IL-8), and regulatory- cytokines (IL-10 and TNF-α), and normalized cytokine scores were computed for each group. Our chosen cytokine scoring systems included individually validated markers by a number of reports in the literature [10–13]. Herein, we included IP-10 within the composite IFN-regulated chemokine score as a marker of the effects of IFN and not to suggest it was specifically upregulated only due to IFN, since many cytokines and chemokines including IP-10 are a matrix of overlapping responses.

### Statistical methods

Medians, minimums and maximums were used to summarize the chemokine score values. Chemokine scores were compared between autoantibody groups using Kruskal-Wallis rank-sum tests. Linear regression models were used to examine the association between changes in muscle disease activity and physician global disease activity VAS according to autoantibody groups and chemokine scores. Interactions between autoantibody groups and chemokine scores were examined. We performed our analysis using the muscle and physician global VAS specifically since Reed et al. [2] previously reported that these measures had the most correlation with IFN score.

## Results

### Baseline characteristics

**Fig. 1:**
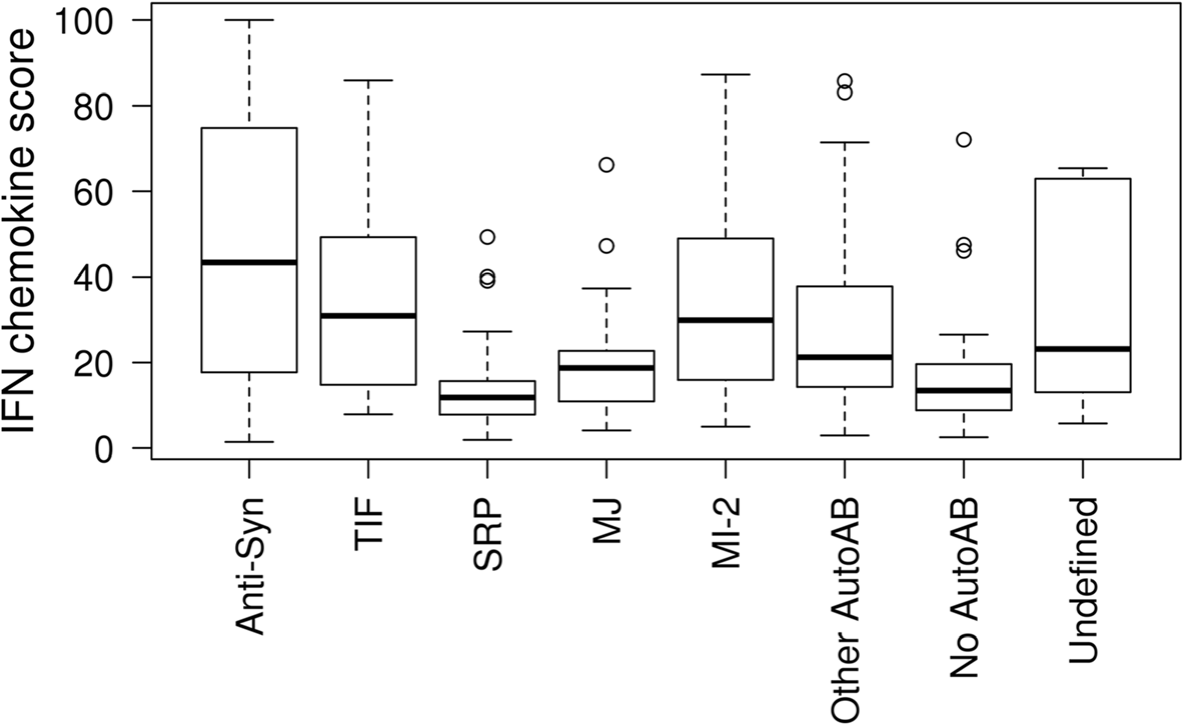
Distribution of cytokines scores at the start of treatment by autoantibodies. Groupings include the individual autoantibodies listed and those individuals who had no autoantibodies detected (no autoantibodies), those with myositis-associated autoantibodies but not those listed (other autoAb) and those with autoantibodies that are not able to be defined (undefined)

Cytokine and chemokine analysis and clinical information were available for 177 of 200 subjects from the RIM trial. Detailed data on subjects’ demographics, baseline disease characteristics, safety and clinical outcomes of the RIM trial were previously reported [1]. Briefly, subjects had longstanding (mean [SD] = 5.4 [6.5] years) and highly active disease as evidenced by the physician global disease activity (mean [SD] = 49 [19] mm), patient global disease activity (mean [SD] = 65 [21] mm) and muscle activity VAS scores (mean [SD] = 46 [22] mm). Subjects had failed a mean of 3.1 immunosuppressive agents in addition to glucocorticoids. [1] We found that IFNCK scores (median values) were higher at baseline in subjects with anti-synthetase (43), TIF1-γ (31) and Mi-2 (30) compared with other autoAb groups (*p* < 0.001) (Table 1, Fig. 1). Regulatory scores were higher at baseline in subjects with anti-synthetase (31) and non-MAA (32) vs. other groups (*p* = 0.01) (Table 1).

**Table 1:** Distribution of cytokines scores at the start of treatment by autoantibodies

Baseline	Anti-synthetase	TIF	SRP	MJ	MI-2	Other AutoAb	No AutoAb	Undefined	*P* value
	(*N* = 28)	(*N* = 19)	(*N* = 21)	(*N* = 18)	(*N* = 25)	(*N* = 24)	(*N* = 33)	(*N* = 9)	
IFNCK^a^	43.3	30.9	11.8	18.7	29.9	21.2	13.4	23.2	<.001
	(1.4, 100.0)	(7.9, 85.9)	(1.9, 49.3)	(4.1, 66.2)	(5.0, 87.2)	(2.9, 85.7)	(2.5, 72.1)	(5.7, 65.4)	
TH1	27.9	23.3	19.6	21.9	21.8	26.4	20.7	22.2	0.070
	(6.1, 80.5)	(13.5, 77.1)	(9.5, 88.9)	(3.8, 80.6)	(17.2, 51.6)	(16.6, 49.0)	(10.2, 76.2)	(12.5, 93.3)	
TH2	11.7	10.8	10.1	10.3	9.5	11.7	10.6	10.9	0.12
	(5.4, 47.8)	(6.8, 52.5)	(2.4, 93.7)	(2.2, 89.8)	(6.5, 21.4)	(7.6, 88.4)	(6.7, 82.8)	(3.5, 34.8)	
TH17	28.1	22.8	22.6	27.6	20.7	24.5	22.4	33.1	0.24
	(13.0, 79.2)	(12.7, 48.9)	(13.5, 62.5)	(8.6, 74.6)	(11.2, 47.8)	(15.4, 53.9)	(8.9, 40.1)	(14.8, 68.5)	
Innate	49.3	31.0	28.4	33.3	31.0	33.6	27.0	37.8	0.001
	(19.1, 74.8)	(15.5, 54.9)	(11.7, 54.4)	(18.1, 71.2)	(17.0, 53.1)	(17.4, 61.6)	(16.2, 58.5)	(18.2, 63.8)	
Regulatory	31.1	23.0	21.0	23.1	19.9	32.2	21.1	19.9	0.010
	(13.0, 75.6)	(10.6, 100.0)	(7.5, 100.0)	(3.5, 82.1)	(15.7, 55.1)	(14.6, 75.1)	(11.6, 76.8)	(15.4, 69.5)	

^a^ *IFNCK* IFN chemokine score

**Fig. 2:**
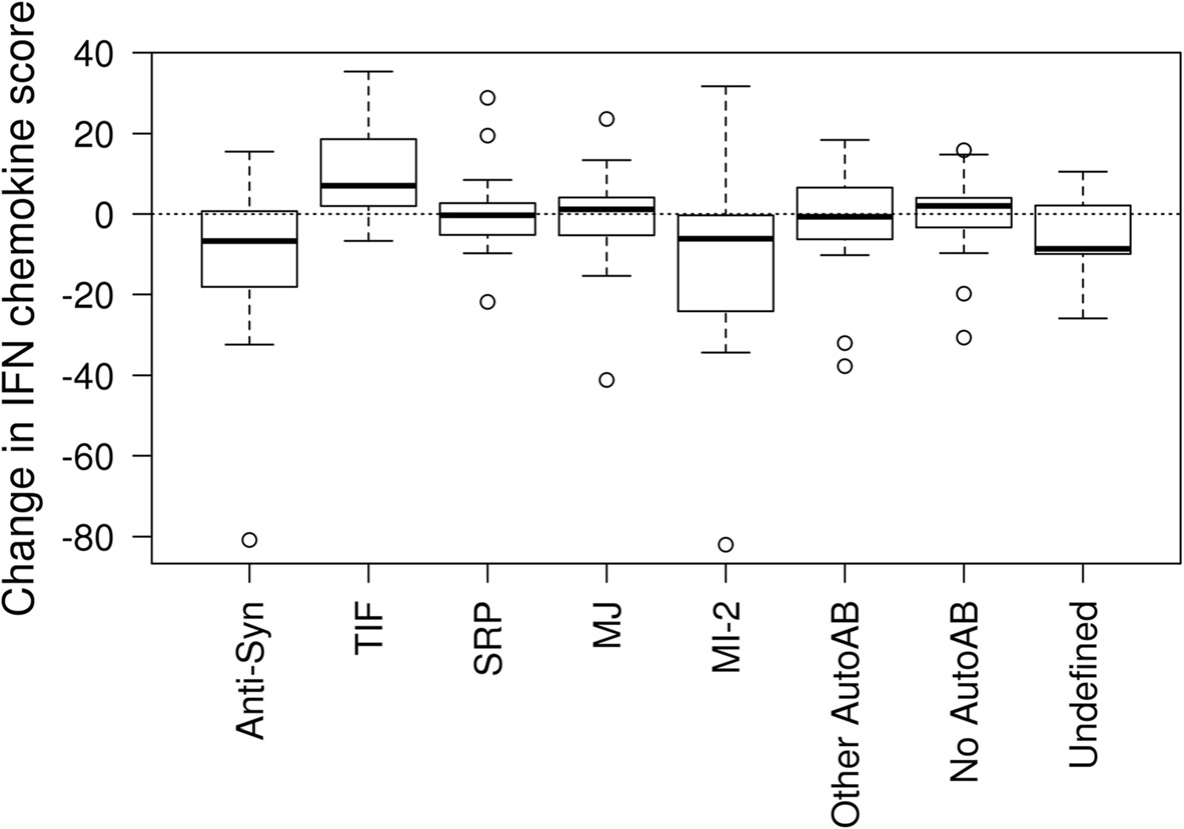
Distribution of cytokines scores of the change from start of treatment to 16 weeks after the start of treatment by autoantibodies

### Changes from baseline in cytokine scores by autoantibodies following treatment with rituximab

We compared the distribution of cytokine/chemokine scores based on autoantibody groups between the baseline and follow-up time points using the Kruskal-Wallis rank sum test to determine if there is additional predictability when these assessments are combined Figure 2. Baseline was defined as the serum cytokine/chemokine scores based on autoantibody groups before initiation of rituximab treatment. No significant improvement in cytokine/chemokine scores based on autoantibody groups was detected at 8 weeks after the start of treatment. However, at 16 weeks after BCD, anti-synthetase and Mi-2 autoAb and “undefined” autoAbs positive subject subgroups had a greater improvement (decrease) in IFNCK scores (−6.7, −6.1 and −8.7, *p* < .001), while TIF1-γ positive subjects worsened by 7.0. The regulatory score improved at 16 weeks in anti-synthetase (−5.8), Mi-2 (−3.4) and non-MAA (−7.2) subjects (Table 2). These three groups stand out when compred to the other 8 groups (*p* = 0.028). Th1 scores also improved in the anti-synthetase, Mi-2, non-MAA and to a lesser extent in the TIF1-γ group at 16 weeks (*p* = 0.039) with the greatest improvement at 24 weeks (*p* = 0.014), suggesting a longer time to improvement if the Th1 score was elevated. The Th17 score remained unchanged.

**Table 2:** Distribution of cytokines scores of the change from start of treatment to 16 weeks after the start of treatment by autoantibodies

Change from baseline to 16 weeks later	Anti-synthetase	TIF	SRP	MJ	MI-2	Other AutoAb	No AutoAb	Undefined	*P* value
	(*N* = 23)	(*N* = 15)	(*N* = 18)	(*N* = 15)	(*N* = 13)	(*N* = 18)	(*N* = 29)	(*N* = 9)	
IFNCK^a^	−6.7	7.0	−0.3	1.2	−6.1	−0.7	2.0	−8.7	<.001
	(−80.8, 15.5)	(−6.7, 35.3)	(−21.9, 28.8)	(−41.2, 23.6)	(−82.0, 31.6)	(−37.7, 18.4)	(−30.6, 15.9)	(−25.8, 10.5)	
TH1	−3.4	−0.9	0.4	0.8	−2.6	−3.6	−0.3	−2.4	0.039
	(−44.9, 34.5)	(−4.9, 34.9)	(−44.0, 63.3)	(−20.7, 32.2)	(−27.8, 3.6)	(−20.7, 17.7)	(−59.8, 41.8)	(−6.0, 60.3)	
TH2	−1.4	0.6	1.1	2.4	0.2	−0.3	1.4	1.3	0.11
	(−9.5, 6.1)	(−17.5, 5.8)	(−7.5, 41.1)	(−7.0, 41.4)	(−5.7, 8.8)	(−51.9, 19.5)	(−69.3, 25.5)	(−5.1, 23.8)	
TH17	−0.0	3.3	3.9	0.0	2.1	0.6	3.0	−1.0	0.50
	(−22.4, 26.8)	(−20.2, 26.7)	(−15.8, 45.7)	(−25.3, 43.2)	(−18.8, 39.1)	(−22.0, 24.5)	(−20.4, 43.0)	(−13.3, 13.8)	
Innate	2.0	3.7	2.7	3.6	−1.0	1.4	7.6	2.1	0.029
	(−21.7, 14.9)	(−7.8, 24.0)	(−6.6, 34.5)	(−20.3, 21.6)	(−24.3, 8.3)	(−14.5, 23.2)	(−16.5, 22.1)	(−20.1, 14.0)	
Regulatory	−5.8	−1.9	−0.7	−0.2	−3.4	−7.2	−1.4	−2.9	0.028
	(−50.3, 17.1)	(−15.4, 15.1)	(−15.4, 55.3)	(−16.6, 25.0)	(−32.2, 9.1)	(−50.0, 24.9)	(−47.0, 45.7)	(−11.7, 12.7)	

^a^ *IFNCK* IFN chemokine score

### Changes in muscle VAS at 16 weeks by conjunction of IFNCK scores and autoAb groups

**Fig. 3:**
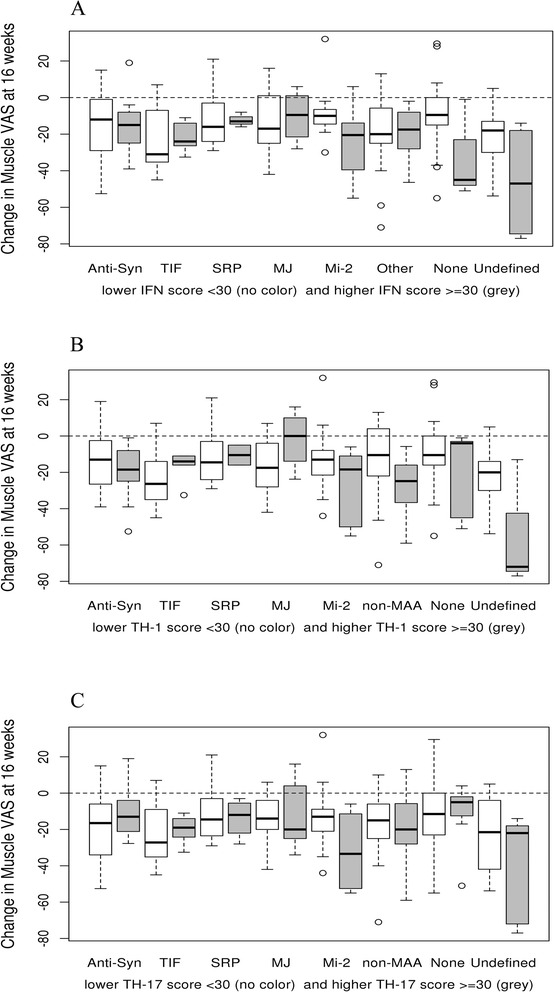
Changes in muscle VAS at 16 weeks by conjuction of serum IFN chemokine scores and autoAb groups. **a** Changes in Muscle VAS at 16 weeks with serum IFNCK score. **b** Changes in Muscle VAS at 16 weeks with TH1 score. **c** Changes in Muscle VAS at 16 weeks with TH17 scores

Regression analyses of clinical improvement were chosen based on previously published measures that correlated with IFNCK scores. Muscle VAS changes at 16 weeks revealed a marginally significant interaction between autoantibody groups and IFNCK scores at baseline (*p* = 0.075 for 7°-of-freedom test for interaction). The model showed that high IFNCK scores at baseline predicted larger improvements in muscle VAS at 16 weeks after treatment among subjects in the Mi-2 autoantibody group (*p* = 0.019), the no autoantibody group (*p* = 0.043) and the undefined autoantibodies group (*p* = 0.024) compared to the anti-synthetase group. To depict the interaction, the changes in muscle VAS at 16 weeks were compared among autoAbs subgroups by dichotomizing the subjects based on IFNCK scores into low (<30) and high (>30) groups (Fig. 3 (a)).

In addition, significant interactions were found for muscle VAS changes at 16 weeks between AutoAb subgroups and the baseline TH-1 (*p* = 0.008) and TH-17 scores (*p* = 0.048). Both interactions indicated larger improvements in muscle VAS at 16 weeks among subjects in the non-MAA and undefined autoantibody subgroups with higher baseline TH-1 and TH-17 scores (Fig. 3 (b & c)). There were no significant associations or interactions among other autoantibody subgroups for muscle VAS. Results for physician global VAS scores were similar to those for muscle VAS, but the interactions between autoantibodies groups and IFNCK, TH-1 and TH-17 scores did not reach statistical significance (*p* = 0.09, *p* = 0.09 and *p* = 0.28, respectively).

## Discussion

We found that biomarker signatures in conjunction with autoAbs prior to treatment help guide response to BCD in refractory myositis. First, we noticed that IFNCK scores were higher at baseline in patients with certain autoAb groups such as anti-synthetase, TIF1-γ and Mi-2. Interestingly, after BCD, patients with (+) anti-synthetase, Mi-2 autoAb (+) patients and “undefined” autoAbs had a greater improvement in IFNCK scores while TIF1-γ (+) patients worsened. Finally we observed that patients with IFNCK high scores in conjunction with the autoAb groups anti-synthetase, Mi-2, non-MAA, and “undefined” autoantibody demonstrated the greatest clinical improvement in terms of muscle VAS. Therefore, results of our current study indicate that autoAbs, especially anti-synthetase, anti-Mi-2, non-MAA, and “undefined” autoAbs in conjunction to IFNCK high scores, are strong predictors of response in rituximab treated myositis patients in the RIM trial. Since commonly associated with immune complexes, a high IFN signature in the absence of defined autoantibodies will more likely suggest antibodies not tested for. Our study is novel since it is the first to demonstrate that subset of autoAbs have a high correlation with interferon chemokine scores.

As previously mentioned, Aggarwal et al. studied the predictability of autoAbs for clinical improvement in patients treated with BCD. His results indicated that autoAbs, especially anti-synthetase (mainly anti-Jo-1) and anti-Mi-2, were the strongest predictors of response in rituximab treated myositis patients in the RIM trial [8]. It is interesting to note that in our study we found that both anti-synthetase and anti-Mi-2 autoAbs in conjunction to IFNCK high scores, were among the strongest predictors of response in rituximab treated myositis patients in the RIM trial. Therefore, this indicates that anti-synthetase and anti-Mi-2 have a strong predictive value for response in rituximab treated myositis patients. In fact, previous studies have shown anti-Mi-2 to be associated with a favorable outcome. In a large cohort of anti-Mi-2 positive patients studied by Hengstman et al., the anti-Mi-2 positive patients had a better treatment response than the control group of patients with myositis [8]. Similarly, in a study by Hamaguchi et al., the prognosis of patients with anti–Mi-2 was favorable [14]. Nevertheless, using anti-Mi-2 in conjunction with IFNCK scores could be more sensitive and specific than using anti-Mi-2 alone since the two together are a stronger predictor of response in rituximab treated myositis patients than anti-Mi-2 by itself.

Anti-synthetase (anti-Jo being the most common and well known) has been heavily studied as a biomarker of myositis disease activity. While the IFNCK score did improve in anti-synthetase (+) patients, the change in global and muscle disease VAS when put in conjunction with high IFNCK scores was moderate. This could suggest that there is not much benefit of using anti-synthetase in conjunction with IFNCK scores. Aggarwal et al. did find that anti-Jo predicts clinical improvement [15]. Nevertheless, it is important to note that the IFNCK score, which helps identify rituximab responsiveness in refractory myositis patients, does improve in anti-synthetase (+) patients.

While Aggarwal et al. found that those with no definable myositis autoantibodies had a worse outcome [15], suggesting that possessing an autoantibody may predict a favorable response, we observed that patients with no autoAbs and “undefined” autoAbs and higher IFNCK scores were among the autoAbs subgroups that demonstrated the greatest clinical improvement. This demonstrates that IFNCK scores could be useful for discriminating patients who will improve from those that will not among those with no or undefined autoantibodies.

## Conclusions

Our study is the first to comprehensively demonstrate that biomarkers in conjunction with autoAbs are major predictive factors of response in myositis patients treated with BCD. This information is useful since biologic therapies such as rituximab have brought improved efficacy in the realm of autoimmune diseases, but their use in clinical practice may be limited by concerns over cost. Predictive models are, therefore, needed to identify those people with autoimmune diseases with the worst potential outcomes, who will benefit most from the use of these drugs. It’s important to bear in mind that prediction methodology should not only be sensitive and specific, but should be simple enough so that they are not limited by their complexity or the need for many biomarkers that will never be routinely measured in the clinic. Therefore, future studies should focus on designing a mathematically weighted matrix which will serve as a predictive score for refractory myositis disease using clinical disease features, interferon gene and chemokines, as well as myositis antibodies in order to predict which patients will or will not respond to rituximab.

## Abbreviations

autoAbs: Autoantibodies
BCD: B cell depletion
CSMs: Core set measures
DM: Dermatomyositis
IFN: Interferon
IFNCK: Interferon chemokine
IIMs: Idiopathic inflammatory myopathies
ILD: Interstitial lung disease
IMACS: International Myositis Assessment and Clinical Studies
MAA: Myositis associated autoantibody
MDAAT: Myositis Disease Activity Assessment Tool
MMT: Manual muscle testing
MSA: Myositis specific autoantibody
PM: Polymyositis
RIM: Rituximab in Myositis Trial
Syn: Synthetase
VAS: Visual analogue scale
